# Liquid Biopsies beyond Mutation Calling: Genomic and Epigenomic Features of Cell-Free DNA in Cancer

**DOI:** 10.3390/cancers13225615

**Published:** 2021-11-10

**Authors:** Arlou Kristina Angeles, Florian Janke, Simone Bauer, Petros Christopoulos, Anja Lisa Riediger, Holger Sültmann

**Affiliations:** 1 Division of Cancer Genome Research, German Cancer Research Center (DKFZ) and German Cancer Consortium (DKTK), 69120 Heidelberg, Germany; a.angeles@dkfz.de (A.K.A.); f.janke@dkfz.de (F.J.); mone-bauer@gmx.de (S.B.); 2 National Center for Tumor Diseases (NCT), 69120 Heidelberg, Germany; petros.christopoulos@med.uni-heidelberg.de; 3 Translational Lung Research Center, German Center for Lung Research (DZL) at Heidelberg University Hospital, 69120 Heidelberg, Germany; 4 Medical Faculty, Heidelberg University, 69120 Heidelberg, Germany; 5 Department of Oncology, Thoraxklinik at Heidelberg University Hospital, 69126 Heidelberg, Germany; 6 Helmholtz Young Investigator Group, Multiparametric Methods for Early Detection of Prostate Cancer, German Cancer Research Center (DKFZ), 69120 Heidelberg, Germany; anjalisa.riediger@med.uni-heidelberg.de; 7 Department of Urology, Heidelberg University Hospital, 69120 Heidelberg, Germany; 8 Faculty of Biosciences, Heidelberg University, 69120 Heidelberg, Germany

**Keywords:** liquid biopsy, cell-free DNA, precision medicine, fragmentomics, epigenomics, DNA methylation, DNA hydroxymethylation, histone modification, nucleosome positioning

## Abstract

**Simple Summary:**

Liquid biopsies provide a non-invasive means to diagnose and profile tumors when tissue is not available. Sequence-based analysis of cell-free DNA (cfDNA) is frequently used to characterize genomic alterations, with a focus on driver mutations or mechanisms of acquired therapy resistance. However, the epigenome of cfDNA also contains additional information about the tumor, which might open new possibilities for clinical applications. Recent highlighted publications are reviewed on the analysis of fragmentation, epigenomic alterations, as well as nucleosome modifications using cfDNA in various cancers. The potential, challenges, and future directions of genomic and epigenomic analysis of cfDNA in oncology are discussed.

**Abstract:**

Cell-free DNA (cfDNA) analysis using liquid biopsies is a non-invasive method to gain insights into the biology, therapy response, mechanisms of acquired resistance and therapy escape of various tumors. While it is well established that individual cancer treatment options can be adjusted by panel next-generation sequencing (NGS)-based evaluation of driver mutations in cfDNA, emerging research additionally explores the value of deep characterization of tumor cfDNA genomics and fragmentomics as well as nucleosome modifications (chromatin structure), and methylation patterns (epigenomics) for comprehensive and multi-modal assessment of cfDNA. These tools have the potential to improve disease monitoring, increase the sensitivity of minimal residual disease identification, and detection of cancers at earlier stages. Recent progress in emerging technologies of cfDNA analysis is summarized, the added potential clinical value is highlighted, strengths and limitations are identified and compared with conventional targeted NGS analysis, and current challenges and future directions are discussed.

## 1. Introduction

The development of advanced genomic technologies has caused an upsurge in methods of cell-free DNA (cfDNA) analysis during the past decade. The potential clinical utility of these novel techniques is huge, ranging from the non-invasive monitoring of infections, to the early diagnosis of graft rejection after solid organ transplantation [1,2,3], detection of fetal aneuploidy in pregnant women [4,5], and a wide array of applications in oncology. The main benefits of cfDNA over tissue analysis for cancer patients are a higher sampling frequency with reduced procedural risk, as well as the ability of each sample to reflect all tumor lesions in the body, thus overcoming the obstacles posed by temporal [6] and spatial [7] tumor heterogeneity. Thus, cfDNA can be used to explore the evolutionary dynamics of cancers across the entire genomic spectrum. Accordingly, circulating tumor DNA (ctDNA) can be used to diagnose and profile tumors at initial diagnosis, monitor disease progression, characterize mechanisms of acquired resistance, identify minimal residual disease (MRD), and discover potential novel therapeutic targets.

cfDNA sources vary in certain physiological or disease contexts. In healthy individuals, the main sources of cfDNA are lymphoid and myeloid cells, consistent with the frequent turnover of hematopoietic lineage cells in the blood [8]. In cancer, the detection and quantification of mutations, CNVs, and aneuploidies from tumor-derived cfDNA have been successfully used to monitor advanced disease and treatment response [9,10,11]. CfDNA concentrations were shown to be elevated in cancer patients (even at localized disease) compared with healthy individuals [12,13]. Multiple studies have also reported the potential of ctDNA analysis in oligoprogression [14] as well as in early detection of disease progression (i.e., lead time), in which detection of tumor mutational clones precedes conventional imaging modalities [15,16,17,18]. However, determining the source of cfDNA (i.e., tumor vs. healthy tissue) remains a prevailing limitation in genomic analyses of cfDNA. Another limitation is that—while it is possible to track tumor progression based on cfDNA single nucleotide variations (SNVs) and copy number variations (CNVs)—definitive identification of a tumor’s tissue-of-origin based on these parameters is not always possible.

Beyond the conventional cfDNA analysis using targeted NGS (tNGS) [19,20], epigenomic changes in tumor tissues also vary during tumor initiation and progression [21]. In addition, DNA methylation and chromatin modifications or transcription factor binding sites have been described to be highly tissue specific [22,23,24,25,26], and could be used to resolve the tissue-of-origin in early cancer detection [27,28,29]. For example, *SEPT9* promoter methylation is a biomarker for the early detection of lung adenocarcinoma and colorectal cancer (CRC) [30,31]. cfDNA fragments also harbor information about the nucleosome occupancy in tissues, which might inform about their cell-of-origin [8]. Still, a major challenge hindering translation into clinical applications is the low abundance of analytes derived from certain tumor types, such as prostate [32], glioblastoma [33], and renal cancers [34], especially in early tumor stages [35].

## 2. Fragmentation Patterns of cfDNA

For many years, it has been known that the size distribution of cfDNA is not random and might contain information about the mechanisms of its release [36]. The cfDNA size peak of ~167 bp reflects the length of the DNA strand wrapped around nucleosomes (147 bp) plus linker DNA fragments of 20 bp. This pattern is generated via caspase-dependent DNA cleavage, which implies that a large fraction of cfDNA is released from cells undergoing apoptosis. However, recent studies suggesting that cfDNA fragmentation depends on certain pathological conditions, such as cancer [37,38,39,40,41], have fueled research on the identification of additional fragmentation mechanisms (Table 1).

### 2.1. Shortening of cfDNA Fragments in Cancer

Jiang et al. [42] demonstrated that the cfDNA fragment length profile in patients suffering from hepatocellular carcinoma (HCC) was shifted towards lower sizes compared with healthy individuals (145 bp). They estimated the fractions of ctDNA based on the prevalence of copy number alterations (chromosome arm-level z-score analysis). Samples with high fractions of ctDNA showed increased proportions of fragments <150 bp, whereas ctDNA content and fragments between 150 and 180 bp were not correlated. When analyzing fragment size distributions of different tumor entities, Mouliere et al. made similar observations, showing that tumors known to shed high amounts of ctDNA (e.g., lung or colorectal cancer) into the bloodstream also presented increased proportions of fragments <150 bp, compared with low ctDNA cancers (e.g., renal cancer, glioblastoma) and to cfDNA from healthy donors [41]. cfDNA shortening was also observed during pregnancy, where DNA derived from the fetus is ca. 20 bp shorter than maternal cfDNA [43,44], and between donor and host cfDNA in organ transplant recipients [45].

The biological and physical processes responsible for cfDNA shortening are not fully understood. Epigenomic processes might play a role, since hypomethylated cfDNA fragments tend to be shorter than hypermethylated DNA [46,47]. Considering that DNA methylation influences nucleosomal packaging [48], hypomethylated cfDNA might be less densely associated with nucleosomes and therefore more susceptible to nucleases.

**Figure 1:**
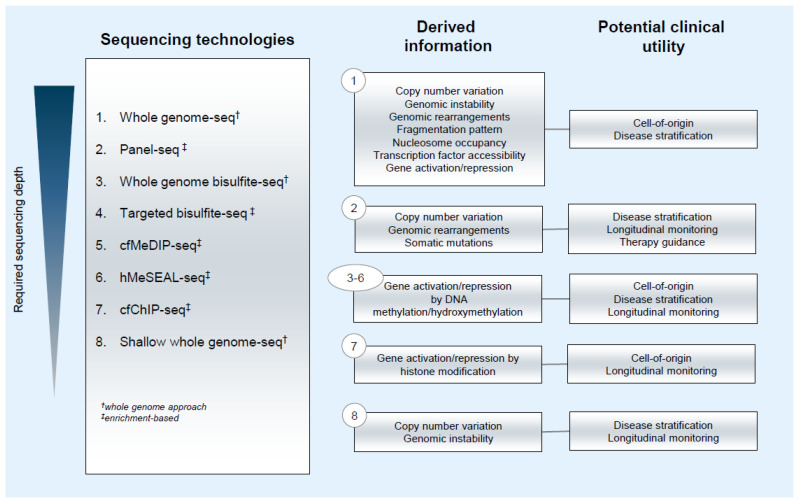
Information output and potential clinical utility of sequencing technologies applied for ctDNA analysis.

Several studies have investigated the shortening of cfDNA for improved tumor detection [37,38,41,49]. For example, Mouliere et al. [41] achieved up to 11-fold ctDNA enrichment by analyzing cfDNA fragments between 90 and 150 bp only. Consequently, they were able to improve the detection of copy number variations (CNV) and single nucleotide variations (SNV). Further studies corroborated the enhanced sensitivity of CNV and SNV detection after selecting short cfDNA fragments using either in vitro or in silico enrichment methods [34,38,41,50,51]. Cristiano et al. [37] applied a different approach in which the length variations of ctDNA were considered in a position-specific manner using shallow whole genome sequencing (sWGS). They compared the fractions of small (100 to 150 bp) to large (151 to 220 bp) cfDNA fragments within 5 Mb bins throughout the genome in samples from 236 healthy donors and 245 patients suffering from various cancers. cfDNA fragments of healthy individuals were highly concordant with and reflected the fragmentation of lymphocytic nucleosomal DNA. The cfDNA fragmentation from patients was much more variable and exhibited regional increases in particular fragment lengths. In a subset of these samples, the degree of fragmentation alterations was correlated to the SNV fractions. This suggested that position-specific alterations of cfDNA size profiles could be used for cancer detection. Using machine learning (ML) on the cfDNA fragmentation profile data, cancer patients could be identified with a sensitivity of 73% and a specificity of 98%. The tissue-of-origin was correctly classified in 61% of cases [37]. Smith et al. applied a random forest algorithm for ctDNA detection based on cfDNA fragmentation features obtained from sWGS of plasma and urine DNA in renal cell carcinoma (RCC) patients [34] and obtained 91.7% prediction accuracy. This result indicates that sWGS is an inexpensive approach which can identify samples for targeted NGS analyses to detect potentially actionable gene-specific alterations (Figure 1).

While double-stranded DNA (dsDNA) library preparation is insensitive to highly degraded cfDNA, single-stranded (ssDNA) libraries can capture such molecules and might provide a better insight into shorter-sized DNA molecules: using ssDNA library preparation and quantitative PCR, a cfDNA population with a significantly shorter fragment length profile (30 to 130 bp) was identified compared with the standard dsDNA method [8,52,53,54]. These data also revealed that circulating DNA does not only associate with nucleosomes but also tends to be occupied by transcription factors (TFs) with size ranges between 20 and 90 bp [54]. In addition, ssDNA sequencing data showed that nucleosome-associated cfDNA presents high numbers of ssDNA breaks that are missed in the preparation of conventional dsDNA libraries. These single-stranded nicks were found to be more frequent in samples of cancer patients compared with healthy individuals, indicating higher nuclease activity in patients [53,54]. The recovery of very short DNA molecules might also improve the detection of mitochondrial cfDNA that revealed promising results in the differentiation between HCC patients and healthy donors [42], or of recipient vs. donor cfDNA after lung transplantation [52]. In contrast, fragments >250 bp present only a minor fraction of the cfDNA repertoire and have received little attention so far. However, one study observed that mutated cfDNA was also enriched for fragments between 250 and 320 bp compared with non-mutated cfDNA fragments [41].

### 2.2. Cancer-Associated Alterations of cfDNA Fragment End Sequences

The ends of cfDNA fragments provide insights into the biology of cfDNA fragmentation and were also shown to have potential clinical implications in cancer patients. Such informative characteristics include the genomic location of the fragment ends [55,56], their sequence context at the 5′-end [39,57,58,59], and single-stranded 5′ protruding (“jagged”) ends [60,61]. Whole genome sequencing (WGS) of cfDNA from pregnant women has shown that cfDNA fragment ends are not equally distributed in the genome [55]. These “plasma DNA preferred ends” were linked to genomic regions with open chromatin, emphasizing that cfDNA is preferentially cleaved at accessible sites of the genome [62]. Since chromatin accessibility differs between cell and tissue types [8,63], the fragment ends of maternal and fetal cfDNA vary considerably and are characteristic for their tissues-of-origin, i.e., hematopoietic cells [42,64,65] vs. placental trophoblasts, respectively [55].

Similar observations were made in HCC patients. Jiang et al. [56] identified cfDNA fragment end coordinates specific for HCC and validated their tumor association in a cohort of 90 HCC patients, 32 healthy donors, 67 chronic hepatitis B virus (HBV) carriers, and 36 patients suffering from liver cirrhosis. Enrichment of tumor-associated jagged ends in cfDNA was only found in HCC patients, further corroborating the concept of tissue- or tumor-specificity of cfDNA ends. Moreover, tumor-derived fragment ends were shorter and correlated to the ctDNA fraction in the same plasma samples.

Chandrananda et al. [57] were the first to show that not only the location but also the sequence context of cfDNA is associated with distinct biological processes. They reported an increased abundance of cytosines at the 5′-ends of cfDNA molecules, which was absent from randomly sheared cellular DNA and mitochondrial cfDNA. This suggests that the 5′-sequence preference is associated with DNA cleavage between nucleosomes. Similar C-end dominance was detected in murine cfDNA samples [58]. The overrepresentation of certain fragment end motifs was linked to the cutting preference of different nucleases, particularly of deoxyribonuclease 1-like 3 (DNASE1L3) [58,59]. DNASE1L3 and DNASE1 are the most abundant DNases in mammalian plasma [66]. While DNASE1L3 cuts chromatin-associated DNA at nucleosomal linker regions, DNASE1-mediated cleavage predominantly occurs at protein-free DNA molecules, generating A-end fragments [40,59]. The expression levels of *DNASE1L3* are significantly reduced in several cancer entities, including breast, lung, colorectal and liver cancer [67]. A study on plasma samples from 34 HCC patients demonstrated that the low expression of *DNASE1L3* in HCC coincides with a reduction of many of the DNASE1L3-associated motifs (e.g., CCCA, CCAG, and CCTG), and an increase in other motifs in these patients (e.g., TAAA, AAAA, and TTTT) [67]. A global increase in the end motif diversity was also observed in the cfDNA from cancer patients, which might be due to the reduced expression of *DNASE1L3*, resulting in altered cfDNA end motif compositions. Elevated end motif diversities were observed in different cancer entities with reduced *DNASE1L3* expression levels (e.g., colorectal and lung cancer) [67].

More recently, cfDNA molecules were found to possess single-stranded 5′ DNA overhangs [60,61]. The presence of these jagged ends is normally masked by the end repair step during conventional sequencing library preparation. To identify jagged ends, overhangs are extended using nucleotides with detectable characteristics. For example, methylated cytosines can be introduced during overhang elongation, and high abundance of methylated CH sites (H: A, T, or C) at DNA fragment ends reflects the “jaggedness” of the original DNA molecules. Analysis of plasma DNA in *DNASE1*-deficient mice showed a decreased abundance of jagged ends compared with wild-type mice, suggesting that the jaggedness of cfDNA is related to *DNASE1* activity [60]. The expression level of *DNASE1* is elevated in HCC tumors compared with normal liver tissue, and cfDNA in HCC patients is more prone to have jagged ends compared with plasma from healthy donors [60]. Fragment end analysis of urinary cfDNA from bladder cancer patients revealed that stage-dependent reduction of *DNASE1* expression coincided with reduced jaggedness of cfDNA compared with controls [61].

The possibility of inferring the tissue-of-origin from cfDNA fragmentation profiles provides a new layer of information that was previously missed. The correlation of the expression levels of nucleases to the abundance of fragment end motifs and jagged end fragments could guide the selection of cfDNA biomarkers in the future.

## 3. Nucleosome Positioning and Chromatin Modifications in cfDNA

### 3.1. Deducing Gene Expression from Nucleosome Positioning and Occupancy

One strategy to identify the cfDNA tissue-of-origin takes advantage of unique nucleosome positioning patterns in different cell types [68,69]. Snyder et al. [8] performed deep sequencing of cfDNA from healthy donors and applied a heuristic approach to generate genome-wide nucleosome occupancy based on the “windowed protection score” (WPS) metric (Table 2). Regions protected from digestion (e.g., by nucleosomes) yield high WPS values, while unprotected DNA regions are marked by low WPS scores. Further analysis of peak-to-peak spacing of nucleosome calls revealed that, in healthy samples, the high proportion of widened nucleosome spacing (~260 bp) is comparable to observations made in cells of lymphoid or myeloid origin. This finding corroborated the notion that hematopoietic cell death is the prevalent source of cfDNA in healthy individuals. Further, the study utilized nucleosome spacing across gene bodies to infer gene expression levels from cfDNA. Open chromatin and active transcription correlated with tighter spacing between nucleosomes. By using nucleosome spacing within gene bodies as a surrogate for gene expression, it was possible to identify cell type specific signatures from deep cfDNA sequencing. The utility of this approach was illustrated by establishing correlations between nucleosome signatures from cfDNA of cancer patients (i.e., small cell or squamous cell lung cancer, colorectal adenocarcinoma, hepatocellular carcinoma, and ductal carcinoma in situ breast cancer) and gene expression signatures of cancer models (i.e., human cell lines and primary tissues) with the same tissues-of-origin. The method succeeded in matching three out of five test cases. While the study revealed the potential of using nucleosome tracks for predicting the cellular origin of tumors, it was limited by the low number of plasma samples tested, which were themselves biased towards harboring high aneuploidy metric scores (all were from stage IV patients).

Building on the insights presented by the previous study, Ulz et al. [63] explored the potential of using nucleosome occupancy at promoters based on WGS of cfDNA, to infer gene expression signatures associated with various cell types. This hypothesis relied on the premise that in actively transcribed genes, the promoter region including sequences of 1 kilobase (kb) downstream of the transcription start site (TSS), is a nucleosome-depleted region (NDR), which allows docking of the transcription initiation machinery. Flanking the NDR are uniformly positioned nucleosomes. Since the DNA at NDRs is not protected once shed into the circulation, sequence coverage of this region is poor. Hence, the read depth at the TSS of active genes is low, with distinct oscillations around the NDR. This is in contrast to higher read depths expected at the TSSs of inactive genes where nucleosome array packaging is denser. Upon comparison of read depth of cfDNA from healthy individuals with publicly available micrococcal nuclease assay datasets, a high concordance (>90%) of cfDNA fragments was found to be derived from white blood cells, validating the utility of this method. Accordingly, Ulz et al. established a metric for gene expression based on read depth coverage of the 2-kb region centered at the TSS and within the NDR defined from −150 bp to +50 bp with respect to the TSS. Read depths of these regions were normalized against relative copy numbers to account for the contribution of copy number changes to absolute read counts. This approach was successful in inferring the 100 most highly and least expressed genes in plasma with an accuracy of 91%. To test the feasibility of the method in clinical samples, the study performed WGS on matched primary tumor and plasma DNA, as well as tumor RNA-seq from two patients with metastasized breast cancer. Integrating these data, it was possible to identify copy number variations (CNVs) to estimate the ctDNA fraction and facilitate accurate predictions of gene expression from promoter read depths. This analysis was limited to regions with high ctDNA allele frequencies, revealing practical caveats of the method, particularly its low prediction accuracy for samples with low tumor load and hence unsuitability in MRD monitoring, as well as in assessing cfDNA from patients with tumors known to shed minimal ctDNA. Nonetheless, the robustness of the method was tested in cfDNA from patients with metastasized cancer of various origins (i.e., colon, prostate, breast, lung; *n* = 426), and 51.6% of the samples were amenable for promoter read depth analysis. Despite the limitations, using NDR quantification at promoter regions expands the repertoire of analyses that can be performed on cfDNA, while also enabling deduction of gene expression from WGS data of cfDNA. A commentary [70] proposed an improvement of the method, which requires deeper sequencing of cfDNA to identify and enrich for specific regions pertinent to the disease context. This way, the sequencing costs can be mitigated while the most informative genomic regions are sufficiently covered.

The work on cfDNA fragmentation patterns was further expanded by Sun et al. upon their development of a novel approach of utilizing tissue-specific open chromatin regions to infer the tissue-of-origin [71]. The method takes cfDNA orientation into consideration and differentiates the upstream (U) and downstream (D) ends of the DNA fragment based on their alignment to the reference genome. The group reproduced the nucleosomal periodicity of ~190 bp as reflected by the depth of coverage at nucleosomes and linkers. Depending on the phasing of the fragment end peaks, it was possible to deduce regions of open chromatin. This was based on the previous observation that open chromatin regions harbor regulatory elements in the absence of nucleosomes which are flanked by well-phased nucleosome arrays. The group developed a metric termed “orientation-aware cfDNA fragmentation” (OCF) value, which measures the differential phasing of U and D fragment ends. Due to the specific chromatin landscapes in different tissue types, distinct ranges of OCF values were accordingly observed in various tissue controls. Finally, OCF analysis was applied to noninvasive prenatal testing, patients with liver pathologies such as liver transplantation and hepatocellular carcinoma, colorectal cancer, and lung cancer patients. This cfDNA assay does not require high sequencing depths (median depth: 3.2×), which differentiates it from the other nucleosomal positioning approaches described above.

### 3.2. Inference of Transcription Factor Binding

In addition to establishing a method to map nucleosome positions from cfDNA sequences, Snyder et al. [8] also deduced the occurrence of TF binding using shorter cfDNA fragments associated with cleavage next to transcription factor binding sites (TFBS), as opposed to longer cfDNA fragments associated with cleavage between nucleosomes. Extending this work, Ulz et al. [72] generated a metric which measures the accessibility of TFBS from cfDNA nucleosome coverage patterns. On initial analysis, read depths at TFBS inform of TF binding, indicative of TF activity specific to certain cell lineages. Accessibility scores were generated for 504 TFs, each with 1000 well-characterized TFBSs. Detection thresholds for TFBS accessibility differences from normal samples were defined, and the measured deviations of accessibility scores were used to validate signature TF activities in prostate, breast, and colon cancer-derived cfDNA. The study also presented three potential clinical applications of TF analysis. First, in a prostate cancer case which transdifferentiated to a treatment-emergent small cell neuroendocrine (t-SCNC) subtype, two plasma samples collected in a 12-month interval showed decreased accessibility of AR binding sites, reflecting the androgen-independent pathology of t-SCNC. Reduced accessibilities were also observed for *HOXB13, NKX3-1*, and *REST*. These observations suggest the possibility of cfDNA TF analysis for molecular tumor subtyping. Second, plasma samples from early-stage colon adenocarcinoma patients (COAD stage I, *n* = 197; stage II, *n* = 280), and from individuals with no cancer diagnosis (*n* = 177) were compared, to assess the resolution limits of TF-based cancer detection. In stage I and II COAD cancers, tumors are still localized and consequently, the tumor fractions from cfDNA for most samples measured less than the detection limit of an established algorithm. However, statistically significant changes in binding by colon cancer-associated TFs were already apparent for early-stage patients compared with healthy controls. Further, upon model training by logistic regression analysis on 504 TFs, using a subset of early-stage COAD samples, the method was able to correctly identify stage I and II patients with precisions of 74% and 84%, respectively. These results highlight the utility of the approach to establish classifiers for predicting early-stage cancers, which is currently a fundamental weakness of cfDNA technologies. Finally, nucleosome positioning studies typically require deep WGS of cfDNA. Notably, Snyder et al. [8] utilized 1.5 billion reads per sample to perform TF footprinting, which would translate to exorbitant clinical costs. In contrast, the current method [72] can perform reliable TF analysis even when sequencing is downsampled to 50 million reads (Figure 1).

### 3.3. Histone Modification of cfDNA Nucleosomes as a Measure of Transcriptional Activity

Sadeh et al. [29] developed a chromatin immunoprecipitation (ChIP) method applicable to cell-free nucleosomes in plasma, followed by sequencing (cfChIP-seq), taking advantage of intact histone marks to define gene expression signatures present in plasma DNA and trace their tissues-of-origin (Table 2). In this study, cfChIP-seq was performed in <2 mL of plasma using antibodies targeting marks of active promoters (H3K4me3 or H3K4me2), enhancers (H3K4me2 or H3K4me1), and gene bodies of actively transcribed genes (H3K36me3). Accordingly, H3K4me3 pulldown of cfDNA from healthy individuals yielded considerably overlapping occupancies with reference H3K4me3 ChIP-seq maps of monocytes and neutrophils, verifying the main contributors of cell-free nucleosomes in healthy plasma. Moreover, cfDNA H3K4me3 enrichment at promoters was consistent with the expression levels of genes in hematopoietic cells based on reference tissue expression data. To test whether cfChIP-seq could predict gene expression changes in patients with underlying pathological conditions, H3K4me3 precipitation was performed on plasma from healthy donors, from patients diagnosed with metastatic CRC, and patients with autoimmune, metabolic, or viral liver disease or with acute myocardial infarction. Using published ChIP-seq data, the authors generated cell type-specific gene expression signatures to be used as references. cfChIP-seq was able to reflect the tissue-of-origin of the most relevant cfDNA contributors in each of these pathologies, based on H3K4me3-inferred transcriptional program activation. The study also challenged the abundance of erythroblast-derived cfDNA in plasma from healthy donors, as previously reported [64,73]. Instead, the authors observed enrichment for megakaryocyte-specific genes, implying that megakaryocytes are major contributors of cfDNA in healthy individuals. For the metastatic CRC cohort, a highly discriminative CRC classifier was generated based on COAD genes from the TCGA dataset (area under the ROC curve [AUC]: 0.94). Furthermore, the CRC signature scores derived from longitudinal cfChIP-seq data showed changes reflecting the disease status of patients at particular points of the therapy lines. The study also explored inter-tumor heterogeneity through cfChIP-seq-derived expression signatures. Five signatures were generated that could stratify metastatic CRC based on gene expression programs and duplication events. However, it remained unclear if these signatures would correlate with the genotypic background of the CRC tumors. Overall, Sadeh et al. developed a nuanced approach for cfChIP-seq analysis that should be extended to larger cohort numbers and perhaps other tumor entities.

Although still in its infancy, conceptually, cfChIP-seq might be used as a surrogate for gene expression and hence be informative of expression signatures that can trace the tissue-of-origin of cfDNA. The potential clinical utility of this strategy includes estimation of tumor load, identification of cellular processes, and assessment of inter-tumor heterogeneity. One advantage of cfChIP-seq over nucleosome positioning approaches is the requirement of significantly fewer sequencing reads. Since ChIP parallels a targeted pulldown approach that reduces coverage of a large part of the genome, informative data can be acquired at lower sequencing costs.

In a proof of principle study, Vad-Nielsen et al. correlated the expression of lung cancer-associated genes with H3K36me3 enrichment in plasma via cfChIP [74]. H3K36me3 pulldown reflected the repression of *SAT2* and *ALK*, and the constitutive activation of *ACGT1* in cfDNA derived from NSCLC patients. The authors also showed that differential expression of *KRT6* paralogs in lung adenocarcinoma (LUAD) and lung squamous cell carcinoma (LUSC), as observed in published immunohistochemical and gene expression data, can be captured by H3K36me3 cfChIP. Despite these interesting data, the study remained exploratory and limited by its small sample size. Moreover, its application for cell-of-origin or tumor subtype identification requires specific markers with distinct and differential expression in the tissue or tumor of interest.

## 4. Epigenomic Modifications of cfDNA

Many studies have characterized epigenomic DNA patterns in tissues of cancer patients and healthy individuals [75,76,77,78], which were also found to be detectable in cfDNA. Their tissue specificity, which is largely retained in cfDNA [79,80] and the high number of alterations in cancer, provide additional information that complements other liquid biopsy approaches (Figure 1).

### 4.1. Methylation Profiling of cfDNA

Recently, several studies used cfDNA methylation to deconvolute the contribution of various cell and tissue types to the cfDNA pool [27,64,65,81]. Sun et al. [65] applied whole genome bisulfite sequencing (WGBS) of cfDNA from 29 HCC patients, four liver transplant recipients, 15 pregnant women and 32 control subjects. Using reference methylomes from 11 tissues and three blood cell types, they calculated that 70 to 90% of plasma DNA is derived from white blood cells, in particular lymphocytes and neutrophils. The contribution of tissue DNA to the cfDNA repertoire varied in accordance with each patient´s physiological or pathological condition: liver transplant recipients and HCC patients demonstrated elevated levels of liver-derived cfDNA, whereas the placental DNA contribution was higher in pregnancy, when compared with the control group. These results demonstrated that tissue-specific methylation can be captured from cfDNA and that the contribution of different tissue types in such data can inform the presence of a tumor and its primary growth site. Moss et al. [64] took advantage of array-based tissue methylation data to generate a reference atlas comprising 25 cell and tissue types, including nine hematopoietic and endothelial cell types. Lymphocytes (12.1%), granulocytes (32.0%), monocytes (10.5%) and erythroid progenitors (29.7%) were identified to be major hematopoietic cells contributing to the cfDNA pool. DNA contributions from vascular endothelial cells (8.6%) and hepatocytes (1.2%) were also observed. The application of their deconvolution algorithm to cfDNA from cancer patients (i.e., breast, lung, and colon; *n* = 11) resulted in correct classification of the majority of cases and identified associations with the response to therapy in prostate cancer patients. In four patients with cancers of unknown primary (CUPs), the deconvolution showed strong tissue-specificity, indicating the tumor´s primary site. Despite these encouraging results, the practicability of this approach was limited by the requirement for a large quantity of cfDNA input material (~100 ng) for array-based methylation analysis.

Apart from the tissue information presented by the cfDNA methylome, the high prevalence of 5mC alterations in cancer can be exploited for sensitive cancer detection and classification, even in early stages [82,83,84,85,86,87,88] (Table 3). Two recent studies [85,86], used an affinity-based methodology, termed “cell-free DNA methylation immunoprecipitation” (cfMeDIP-seq), to enrich and sequence methylated regions from plasma DNA. cfMeDIP-seq interrogates methylation events on a genome-wide scale and was previously demonstrated to sensitively detect and classify various cancer entities [89]. The method only requires minute amounts (1 to 10 ng) of cfDNA input material and is therefore potentially suitable in a clinical setting. Nassiri and Nuzzo et al. [85,86] used cfMeDIP-seq data to train ML algorithms for the detection and classification of intracranial tumors as well as RCC from plasma and urinary cfDNA. Due to their low ctDNA shedding capacity, both tumor types are difficult to detect using liquid biopsies [9,90]. cfMeDIP-seq accurately detected intracranial tumors (*n* = 60) in a cohort of 447 plasma samples (AUC = 0.99), comprising eight tumor types and individuals without cancer [85]. Furthermore, common primary intracranial tumors (i.e., hemangiopericytoma, meningioma, low-grade glial-neuronal tumors, and gliomas; *n* = 161), which are otherwise challenging to discern by magnetic resonance imaging, could be distinguished based on cfMeDIP-seq data. An ML classifier trained on cfDNA-based cfMeDIP-seq data from 97 individuals (*n* = 69 RCC patients, *n* = 15 urothelial bladder cancer patients (UBC), *n* = 133 controls) separated RCC samples from the other two subgroups (RCC vs. controls: AUC = 0.99; RCC vs. UBC: AUC = 0.98). Urinary cfDNA-based classification (*n* = 30 RCC patients, *n* = 15 controls) could also distinguish RCC from control samples (AUC = 0.86) [86], albeit with lower accuracy. Of note, both plasma- and urine-based analyses included stage I and II RCC patients, further highlighting the practicality of cfMeDIP-seq in challenging clinical scenarios. Moreover, successful methylation analysis of urinary cfDNA demonstrates the utility of urine for genomic [34,91,92,93], as well as epigenomic analysis. DNA methylation marks have been detected in urine sediment in various cancer types such as bladder cancer [94], endometrial cancer [95], and prostate cancer [96]. Additionally, urinary cfDNA equally offers the opportunity for DNA methylation analysis in colorectal cancer [97], bladder cancer [98], and RCC [34].

Combining genomic and epigenomic analysis in assessing methylation status, CNA and cfDNA fragment analysis via shallow-depth bisulfite sequencing of urinary cfDNA, Cheng et al. were able to detect bladder cancer with overall sensitivity of 93.5% and specificity of 95.8% [98].

Another way to leverage the high sensitivity of methylation analyses from cfDNA is the utilization of targeted bisulfite sequencing panels. Here, the large number of publicly available reference methylomes paired with the understanding of the different cell types contributing to the plasma DNA pool is of substantial value. Several studies used tissue- and blood cell-derived data to generate cancer-specific methylation sequencing panels. For instance, Moss et al. [84] designed a breast unique biomarker panel interrogating the methylation status of three genomic loci. The average signal of these markers could distinguish breast cancer patients (metastatic: *n* = 17; localized: *n* = 30) from healthy donors (*n* = 64) with high sensitivity and specificity (80% and 97%, respectively). In longitudinal plasma samples, the breast-specific methylation signature was also indicative of therapy response and the presence of residual disease. Similarly, a CRC-specific methylation panel, covering nine marker regions, demonstrated high accuracy for the detection of CRC (AUC = 0.96) [99]. Chen et al. [88] designed a larger sequencing panel (10,613 CpGs in 477 genomic regions) to allow simultaneous detection of multiple cancer entities. Training a logistic regression classifier on the panel-based methylation data, they predicted the presence of disease across five tumor types (i.e., lung, colorectal, liver, stomach, and esophagus) with a sensitivity of 88%. Furthermore, they demonstrated that their tumor detection strategy might be eligible in a cancer screening scenario. In 143 asymptomatic individuals who were later diagnosed with cancer, the assay detected the disease up to four years prior to the clinical diagnosis (sensitivity: 95%).

The Circulating Cell-free Genome Atlas (CCGA) study is a large scale (*n* = 8584 cancer patients; *n* = 6670 controls) ongoing clinical trial that aims to establish cfDNA sequencing for the detection and localization of multiple cancer entities. In the first part of the CCGA study, the interrogation of genome-wide methylation patterns (WGBS) outperformed the assessment of CNVs and SNVs by whole genome or targeted sequencing [100,101]. The second CCGA sub-study used a bisulfite sequencing panel (1.1 million CpGs in 103,456 regions) to detect and classify more than 50 primary tumor types in 2482 untreated cancer patients (all stages) and 4207 individuals without cancer. At a specificity of 99.3%, the test yielded a sensitivity of 43.9% to detect cancers; the tissue-of-origin was correctly classified in 93% of cancer cases [83]. The third part of the CCGA study is a large clinical validation of this assay [102]. The multi-cancer early detection (MCED) validation study included 4077 participants in an independent cohort. The resulting specificity of the test for cancer signal detection was 99.5%, and overall sensitivity was 51.5%. Cancer signals were detected across >50 cancer types, and as expected, the test sensitivity increased with disease. Building on the success of the MCED, large clinical programs are ongoing to evaluate the performance in a screening population, assess clinical implementation and safety [103,104,105].

### 4.2. 5-hydroxymethylation Profiling of cfDNA

5mC can be reverted to its unmodified state by oxidation steps catalyzed by the ten-eleven translocation (TET) enzymes [106,107,108,109]. Despite its low abundance (5mC: 3–4% vs. 5hmC: 0.1–1% of all cytosines [110]), 5hmC is more than an intermediate state during DNA demethylation. 5hmC abundances can remain stable over several cell divisions and are associated with the activation of nearby genes [111,112]. 5hmC residues accumulate at borders of CpG islands and prevent 5mC from spreading inside a hypomethylated region [113,114,115,116,117]. Unlike 5mC, absolute 5hmC abundances vary considerably between different tissue types [118], which might allow a more precise inference of the tissue-of-origin using 5hmC as a biomarker.

Altered hydroxymethylomes have been identified in all human cancer entities. Genome-wide reduction of 5hmC levels is a feature shared by most cancers [119,120] and can be attributed to the impaired activity of TET enzymes, either through inactivating TET mutations or mechanisms inhibiting the catalytic activity of TET (e.g., unavailability of co-factors or mutations in *IDH1/2* genes) [120,121,122]. In recent years, the analysis of 5hmC from cfDNA has gained traction (Table 3). Song et al. [123] developed a highly sensitive methodology capable of selective enrichment for hydroxymethylated DNA groups, termed “hydroxymethylation selective chemical labeling” (hMeSEAL). hMeSEAL has been widely applied for cfDNA-based 5hmC profiling in various tumor entities [124,125,126,127,128,129,130,131,132]. In a proof-of-concept study, hMeSEAL was used to profile 5hmC changes in 49 plasma samples from cancer patients. In lung cancer, global 5hmC levels decreased from low-stage to metastatic tumors. HCC patients could be successfully differentiated from healthy donors and HBV-positive subjects based on 5hmC levels within gene bodies. Loci identified to be hydroxymethylated in HCC contained several genes with increased expression in liver tissue. This observation was in line with the activating effect of 5hmC on transcription and supports the concept of 5hmC in cfDNA as a surrogate for non-invasive inference of gene expression. Furthermore, longitudinal monitoring of 5hmC patterns in HCC patients, following surgical tumor resection, indicated remission or progression of the disease. Finally, ML algorithms detected and differentiated lung, liver, and pancreas cancers based on their 5hmC profiles with prediction accuracies of up to 90% [130]. These promising results were corroborated by several successive studies applying hMeSEAL on multiple cancer types (i.e., B-cell lymphoma, lung, liver, colorectal, gastric, esophageal, breast, prostate, and thyroid cancer). The genome-wide 5hmC profiles were used to derive biomarker panels for cancer classification, stage prediction or early diagnosis [124,125,129,130,131,132].

## 5. Combinatorial Biomarkers for cfDNA Analysis

Epigenomic changes are readily observed at early stages of tumor development [30,133], opening up their potential as diagnostic tools for early cancer detection, risk stratification and MRD. Nevertheless, many liquid biopsy-based assays suffer from reduced sensitivity due to low levels of ctDNA. One promising way to mitigate this obstacle and to increase the detection success is to screen for multiple targets and combine multi-analyte analysis.

Cai and colleagues [126] combined a 5hmC signature (based on 64 loci) with existing diagnostic protein markers (AFP and DCP) to differentiate 135 HCC patients from 165 healthy donors and 62 liver cirrhosis patients. Their diagnostic score (“HCC score”), based on the two marker types, identified HCC patients with an AUC = 0.93 and was correlated with the TNM classification of the tumors. Moreover, the HCC score was indicative of recurrence following surgical tumor resection, and reflected the dynamics of tumor burden in patients with long-term follow-up plasma samples. Another study combined hMeSEAL-based 5hmC with cfMeDIP-seq-based 5hmC profiling. The combination of these biomarkers improved the diagnostic power for the detection of pancreatic cancer compared with using 5mC or 5hmC marker panels alone [127].

Peneder et al. [134] performed a multimodal analysis of cfDNA to overcome the obstacle of low mutational burden in paediatric patients with predominantly Ewing Sarcoma (EwS). Applying deep WGS on 263 cfDNA samples from patients with EwS (*n* = 95), other pediatric sarcomas (*n* = 31), and healthy controls (*n* = 22), they combined copy number alterations, the presence of the *EWS-ETS* fusion oncogene and cfDNA fragment size distributions. The integrated genetic analysis of CNVs and the detection of the *EWS-ETS* fusion gene served for the quantification of the tumor content in cfDNA. Similar to previously mentioned reports, they found that tumor patients harbored a significantly higher proportion of shorter cfDNA fragments <150 bp compared with healthy controls. Combining fragment and genetic analysis, this higher proportion of smaller cfDNA fragments was equally detectable in patient samples in which evidence for ctDNA was missing or only the *EWS-ETS* fusion gene but no CNVs were detected. Fragment size selection further improved CNV analysis and supported the evaluation of CNV dynamics over the disease courses in a subset of the patients. cfDNA samples from EwS patients showed particular position-dependent short to long fragment (S/L) ratios in genome-wide 100-kb bins. By aligning bins with a higher proportion of shorter fragments to regions with known epigenetic and transcription-regulation alterations, an enrichment of regions with EwS-associated open chromatin was found. Finally, based on a ML algorithm and the presented fragmentation analysis methods, cfDNA from EwS patients could be distinguished from healthy controls with high sensitivity and specificity. This study highlighted opportunities for data integration by applying several assays on the same starting material (i.e., cfDNA).

## 6. Challenges and Future Directions

Liquid biopsies are becoming increasingly important in the serial profiling and individualized management of malignant diseases. At present, clinically-approved ctDNA assays act as companion diagnostics that facilitate therapy guidance based on genetic alterations including SNVs, amplifications, insertions/deletions, and translocations [135]. Despite their clinical relevance, the information that can be gained from cfDNA could be significantly enhanced by including epigenomic analyses, as outlined here. One step in this direction is represented by low throughput ctDNA methylation assays, which survey the epigenomic status of validated biomarkers in bladder, breast, colorectal, cervical, lung, and prostate cancers [136] with the intention of disease detection and therapy response prediction. Another advantage of epigenomic ctDNA analyses, particularly of those that generate 5mC and 5hmC data, is the declining sequencing cost. At the appropriate sequencing depth, relevant methylation or hydroxymethylation biomarkers can be identified in a single reaction. The overall cost of these assays can be further reduced through the design of hybridization probes to capture regions of interest. Several cfDNA technologies presented here involve enrichment of genomic targets through antibody- (e.g., MeDIP-seq, ChIP-seq) or chemical affinity-based (e.g., hMeSeal-seq) approaches. As a consequence of target enrichment, moderate sequencing depths of precipitated DNA are sufficient. In contrast, mutation analysis requires high on-target coverage for ctDNA detection and therefore high cost. Furthermore, the epigenomic approaches reviewed here do not necessitate high amounts of input DNA, allowing their application even when cfDNA concentrations are low (e.g., in patients with localized disease).

There are still a number of challenges that must be met prior to a clinical translation of epigenomic ctDNA analyses. While many proof-of-principle studies have shown that fragmentomics, nucleosome positioning, and ChIP-seq of cfDNA can identify the cell(s)-of-origin and possibly differentiate disease stages, there is still a lack of validation of these methods in larger multicenter clinical studies. Another challenge lies in the high quantities of non-tumor cfDNA in cancer patients, which is mostly derived from leukocytes. Extensive bioinformatic expertise is required to discern epigenomic cancer markers from the non-tumor background. Considerable opportunities beyond the epigenomic analysis of cfDNA reside in the inclusion of other molecular analytes from blood or plasma, such as circulating tumor cells (CTCs), mRNA, miRNA, and extracellular vesicles. CTCs in particular have been utilized to demonstrate intrapatient heterogeneity which could explain therapy resistance [137], and to explore the biological mechanisms underlying cancer cell dissemination [138]. CTC quantification has also been shown to be informative of therapy response in breast [139], prostate [140], and bladder [141] cancers. The analysis of other body fluids (e.g., urine, saliva, CSF) could further improve patient management.

## 7. Conclusions

Liquid biopsies in cancer have advanced from DNA mutations to the epigenome. In contrast to somatic genetic mutations, epigenetic features are more dynamic, with the potential of closely reflecting recent physiological alterations. Although challenges remain, the low amounts of DNA required, the large number of possible markers, and the comparatively low per-sample cost are substantial advantages of epigenomic analyses. The integration of different non-invasive markers opens new prospects and augments current established methods for cancer patient stratification, treatment decision and response prediction, timely identification of minimal residual disease and tumor recurrence, as well as early detection.

## Figures and Tables

**Table 1:** Fragmentomic features of cfDNA in cancer.

cfDNA Fragment Feature	Method	Analyte	Control Cohort	Cancer Entity Tested	Result	Reference
Fragment length shortening	WGS	Plasma	Healthy controls (*n* = 32), HBV (*n* = 67), liver cirrhosis (*n* = 36)	HCC (*n* = 90)	Short cfDNA fragments preferentially carry tumor-associated CNVs	[42]
Fragment length shortening	sWGS	CSF	n/a	Glioma (*n* = 13)	Detectable CNVs in CSF correlates with higher abundance of short (<145 bp) cfDNA fragments	[51]
Fragment length shortening	tNGS; ddPCR	Plasma	n/a	Melanoma (*n* = 8), CRC (*n* = 3), and pancreatic (*n* = 2) cancer	2-fold enrichment of SNVs in short (<142 bp) cfDNA fragments	[38]
Fragment length shortening	sWGS; tNGS	Plasma	n/a	High-grade serous ovarian cancer (*n* = 13)	Up to 11-fold enrichment of the mutated cfDNA fraction in short fragments (90 to 150 bp)	[49]
Multiple fragment size features	sWGS; tNGS	Plasma	Healthy controls (*n* = 144)	Multiple cancerentities (*n* = 200)	Enhanced CNV/SNV detection by short fragments; fragmentation feature-based cancer classification (AUC = 0.99)	[41]
Position-specific fragment length variations	WGS	Plasma	Healthy controls (*n* = 245)	Multiple cancer entities (*n* = 236)	Cancer detection based on genome-wide cfDNA fragmentation profiles (AUC = 0.94)	[37]
Preferred end coordinates	WGS	Plasma	Healthy controls (*n* = 32), HBV (*n* = 67), liver cirrhosis (*n* = 36)	HCC (*n* = 90)	Cancer classification based on fragments at tumor-associated preferred end coordinates (AUC = 0.88)	[56]
Fragment end motifs	WGS	Plasma	Healthy controls (*n* = 38), HBV (*n* = 17)	HCC (*n* = 34)	Increased fragment end motif diversity in patients compared with controls	[67]
Jagged ends	WGBS	Plasma	Healthy controls (*n* = 8), HBV (*n* = 17)	HCC (*n* = 34)	Cancer classification based on the increased jaggedness in cancer patients	[60]
Jagged ends	WGBS	Urine	Healthy controls (*n* = 39)	Bladder cancer (*n* = 43)	Cancer classification based on the decreased jaggedness in cancer patients	[61]

AUC, area under the curve; CNV, copy number variation; CRC, colorectal cancer; CSF, cerebrospinal fluid; ddPCR, digital droplet PCR; HBV, hepatitis B virus; HCC, hepatocellular carcinoma; sWGS, shallow whole genome sequencing; tNGS, targeted next-generation sequencing; WGB, whole genome bisulfite sequencing; WGS, whole genome sequencing.

**Table 2:** Histone modifications and nucleosomal positioning in cfDNA from plasma.

Cf Nucleosome Feature	Method	Control Cohort Used for Method Establishment	Cancer Entity Tested	Result	Reference
Nucleosome positioning	Evaluation of windowed protection score (WPS) using deep WGS	Pooled (*n* = 1) and individual cfDNA (*n* = 2) from healthy controls	Small and squamous cell lung cancer, colorectal adenocarcinoma, hepatocellular carcinoma, ductal carcinoma in situ breast cancer	Matched 3 out of 5 cancer test samples to a reference cell-of-origin model	[8]
Nucleosome depleted regions	Analysis of promoter read depth using deep WGS	cfDNA from healthy controls (*n* = 104)	Colon (*n* = 128), prostate (*n* = 139), breast (*n* = 125), lung (*n* = 31) cancers	Identified expressed cancer driver genes from cfDNA in regions with copy number gains	[63]
Nucleosome phasing	Analysis of differential phasing of upstream and downstream cfDNA fragments using WGS	Pooled cfDNA (*n* = 32) from healthy controls	Hepatocellular carcinoma (*n* = 90), colorectal (*n* = 11), and lung (*n* = 9) cancers	Positive correlation of nucleosome phasing between cell-of-origin and patient cfDNA	[71]
Nucleosome footprinting	Assessment of transcription factor accessibility score using WGS	cfDNA from healthy controls (*n* = 24)	Prostate (*n* = 8), breast (*n* = 2), colon (*n* = 1) cancers	Identified cell lineage reprogramming in prostate cancer; identified increased accessibility by tumor entity specific TFs in breast and colon cancer samples	[72]
Histone modifications	cfDNA ChIP-seq	cfDNA from healthy controls (*n* = 61)	Metastatic colorectal cancer (*n* = 56)	Colorectal cancer classifier was established based on histone modification occupancy-inferred expression; longitudinal monitoring reflected the clinical status of patients	[29]
Histone modifications	cfDNA ChIP-qPCR	n/a	Non-small cell lung cancer (*n* = 14)	Correlation of H3K36me3 occupancy and gene expression in lung cancer associated genes	[74]

WGS: whole genome sequencing.

**Table 3:** Epigenomic and hydroxymethylation alterations of cfDNA in cancer.

cfDNA Modification	Method	Analyte	Control Cohort	Cancer Entity Tested	Result	Reference
5mC	Illumina 450k methylation array	Plasma	Healthy control cfDNA (*n* = 105) combined to 8 pools	Colon (*n* = 4), lung (*n* = 4), breast (*n* = 3) cancer, and CUP (*n* = 4)	Tissue-of-origin deconvolution from cfDNA agrees with clinical findings	[64]
5mC	Affinity-based profiling (cfMeDIP)	Plasma	Healthy controls (*n* = 62)	Multiple cancer entities (*n* = 189)	Robust detection and classification across various cancer types (AUC = 0.91–0.98 depending on entity and stage)	[87]
5mC	Affinity-based profiling (cfMeDIP)	Plasma	Various extracranial tumors and healthy controls (*n* = 387)	Multiple intracranial tumor types (*n* = 220)	Accurate detection (AUC = 0.99) and discrimination of common intracranial tumor types (AUC = 0.71–0.95 depending on the type of brain tumor)	[85]
5mC	Affinity-based profiling (cfMeDIP)	Plasma; urine	Healthy controls (*n* = 133 plasma samples; *n* = 15 urine samples)	RCC (*n* = 69 plasma samples; *n* = 30 urine samples), UBC (*n* = 15)	Detection of RCC patients (plasma AUC = 0.99; urine AUC = 0.86) differentiation between RCC and UBC (plasma AUC = 0.98)	[86]
5mC	Targeted bisulfite sequencing	Plasma	Healthy controls (*n* = 64)	Localized (*n* = 30) and metastatic (*n* = 17) breast cancer patients	Cancer detection and therapy surveillance based on a breast-unique 3-marker methylation panel	[84]
5mC	Targeted bisulfite sequencing (PanSeer)	Plasma	Asymptotic individuals (*n* = 414)	Cancer patients (*n* = 223) and asymptotic individuals that were later diagnosed with cancer (*n* = 191)	Cancer detection (up to four years prior to diagnosis) across five cancer entities using a methylation panel covering 447 genomic loci	[88]
5mC	Targeted bisulfite sequencing	Plasma	Healthy controls (*n* = 4207)	>50 cancer entities (*n* = 2482)	Cancer detection in >50 entities with a specificity of 99.3% and accurate tissue-of-origin prediction in 93% of cases	[83]
5hmC	Affinity-based profiling (hMeSEAL)	Plasma	Healthy controls (*n* = 8), HBV (*n* = 7)	Lung (*n* = 15), pancreatic (*n* = 7), gastric (*n* = 5), breast cancer (*n* = 4), HCC (*n* = 10), CRC (*n* = 4), and glioblastoma (*n* = 4)	Detection of disease/stage specific 5hmC changes capable of differentiating lung, liver, and pancreatic cancers	[130]
5hmC	Affinity-based profiling (hMeSEAL)	Plasma	Healthy controls (*n* = 243)	Pancreatic cancer (*n* = 64)	Classification of pancreatic cancer patients based on 5hmC features (AUC = 0.88)	[129]
5hmC	Affinity-based profiling (hMeSEAL)	Plasma	Healthy controls (*n* = 177)	Esophageal cancer (*n* = 150)	5hmC signature-based detection of esophageal cancer (AUC = 0.96)	[131]

AUC, area under the curve; CUP, cancer of unknown primary; HCC, hepatocellular carcinoma; RCC, renal cell carcinoma; UBC, urothelial bladder cancer.
